# Middle Ear Neuroendocrine Tumor Mimicking As Chronic Otitis Media

**DOI:** 10.7759/cureus.42296

**Published:** 2023-07-22

**Authors:** Yuvenesvary Sukumaran, Yazid Pol Ong, Loong Siow Ping, Cheng Ai Ong, Prepageran Narayanan

**Affiliations:** 1 Department of Otorhinolaryngology – Head and Neck Surgery, Queen Elizabeth Hospital, Kota Kinabalu, MYS; 2 Department of Otorhinolaryngology – Head and Neck Surgery, University of Malaya, Kuala Lumpur, MYS

**Keywords:** carcinoid tumor, chronic otitis media, middle ear adenoma, neuroendocrine neoplasm, middle ear tumors

## Abstract

Neuroendocrine neoplasms (NEN) of the head and neck are a rare and diverse group of tumors. Here, we report a case of a 40-year-old man presenting with symptoms resembling chronic left otitis media, including left ear otorrhea, otalgia, and reduced hearing. Otoscopic examination revealed a whitish mass located behind the tympanic membrane. The patient underwent examination under anesthesia and left cortical mastoidectomy, and a histopathological examination of the middle ear biopsy indicated the presence of an epithelial tumor with neuroendocrine differentiation, suggestive of middle ear adenoma. A staging CT scan performed three months after the mastoidectomy showed a hypodensity in the middle ear cavity, with no significant bony erosion, which could potentially indicate a residual or recurrent tumor. Consequently, a radical mastoidectomy was performed. The histopathological examination confirmed the presence of middle ear adenoma with neuroendocrine differentiation.

## Introduction

Middle ear adenoma is a benign tumor, but there is a risk of recurrence and malignant transformation. It is a rare disease, with approximately 150 cases published in the literature to date [1]. Middle ear adenoma is estimated to account for less than 2% of all middle ear tumors. Neuroendocrine neoplasms (NEN) in the middle ear are particularly uncommon, as epithelial cells with neuroendocrine features typically do not reside there [2]. The gastrointestinal tract and lungs are the most frequent locations for NEN [3]. Previous studies have proposed that middle-ear neuroendocrine tumors originate from neural crest cells [4]. The first reported case of a middle ear adenoma, referred to as an “adenomatous tumor,” was documented by Derlacki and Barney in 1976. In 1980, Murphy et al. described the first case of a “carcinoid tumor” in the middle ear, naming neuroendocrine differentiation resembling an adenomatous tumor [5]. Various terms have been used in the literature to describe this tumor, including ceruminoma, ceruminous adenoma, adenomatous tumor, adenocarcinoid, and amphicrine tumor [6]. There is ongoing debate regarding the histopathological features, with some authors suggesting that middle ear carcinoid tumors and adenomas represent distinct entities, while others consider them part of a continuum [5-7]. In an effort to address this controversy, the term “mixed epithelial neuroendocrine tumor” (MENET) was proposed as a more accurate designation [8]. We present the case of a 40-year-old male who was referred to our hospital with chronic left ear discharge, resembling chronic otitis media. Subsequently, he underwent surgery, histopathological examination confirmed the diagnosis of middle ear adenoma. The clinical presentation, as well as the otoscopic and radiologic findings, are nonspecific for middle ear adenomas. Definitive diagnosis relies on histologic and immunohistochemical examination. Establishing the correct diagnosis is crucial for providing appropriate surgical treatment and preventing potential recurrence. Therefore, it is important for ENT surgeons to possess sufficient knowledge regarding this pathology and its differential diagnosis.

## Case presentation

**Figure 1:**
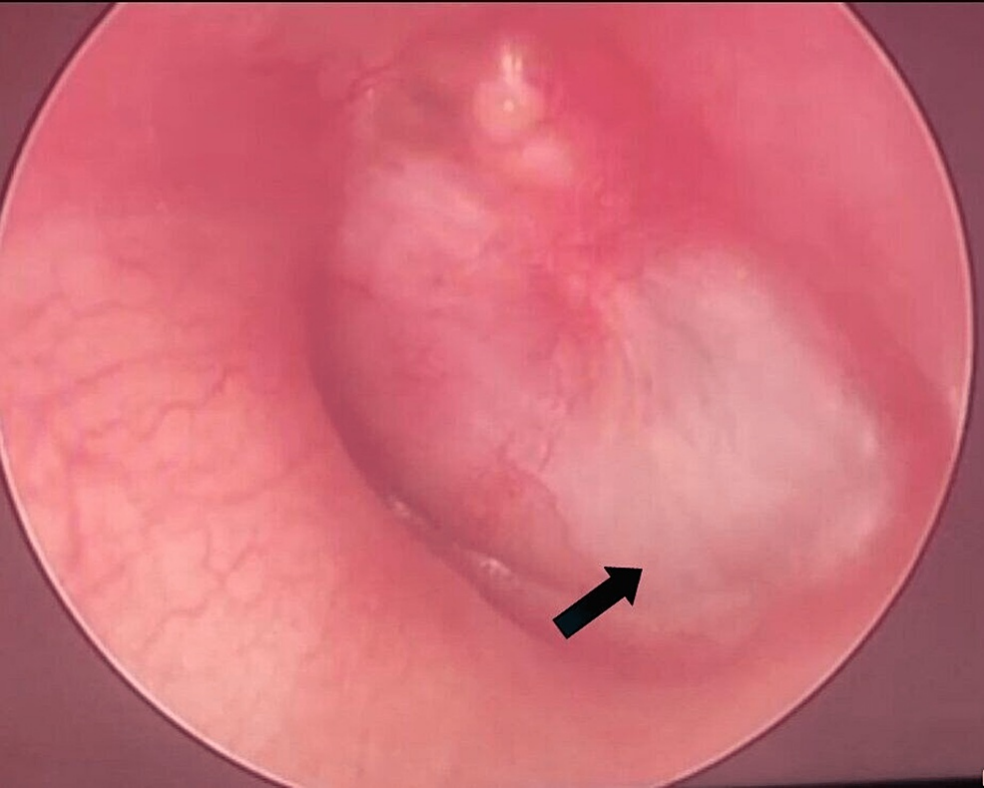
Intraoperative finding showing abnormal bony component at the anterior inferior part of left middle ear

A 40-year-old man with underlying hypertension presented with a nine-month history of left otorrhea, ear blockage, otalgia, and reduced hearing. He denied experiencing tinnitus, vertigo, facial weakness, or fever. Initial examination revealed signs of left otitis media, including the identification of pus in the middle ear and a bulging tympanic membrane bulging. He was initially treated with oral antibiotics for a duration of one week. However, the left ear blockage and pus discharge persisted. The initial hearing assessment showed severe to profound mixed hearing loss in the left ear, with a type B tympanogram. Additionally, there was mild sensorineural hearing loss at high frequency with a type AD tympanogram in the right ear. Subsequent otoscopic examination demonstrated resolved otorrhea and visualization of an opaque mass behind the tympanic membrane (Figure 1). A high-resolution computed tomography (HRCT) was performed, revealing calcification in the middle ear. Consequently, an examination under anesthesia and left cortical mastoidectomy were carried out with intraoperative findings indicating the presence of a bony mass at the medial aspect of the middle ear cavity and unhealthy tissue behind the tympanic membrane.

**Figure 2:**
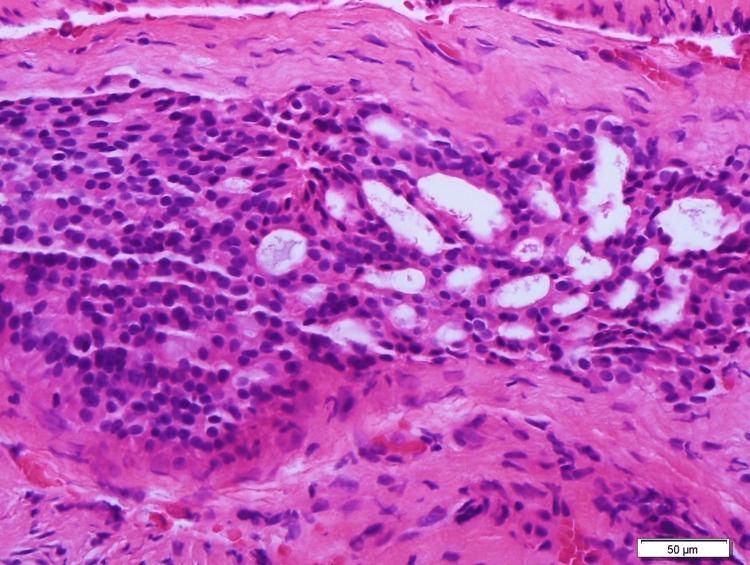
Section from anterior epitympanum show neoplastic cells arranged in cribriform and glandular patterns. These neoplastic cells exhibit uniform, oval to round hyperchromatic nuclei with pale eosinophilic cytoplasm. Mitotic figures are hardly seen. No necrosis, perineural invasion or lymphovascular invasion is observed (x100 and x400 magnification).

**Figure 3:**
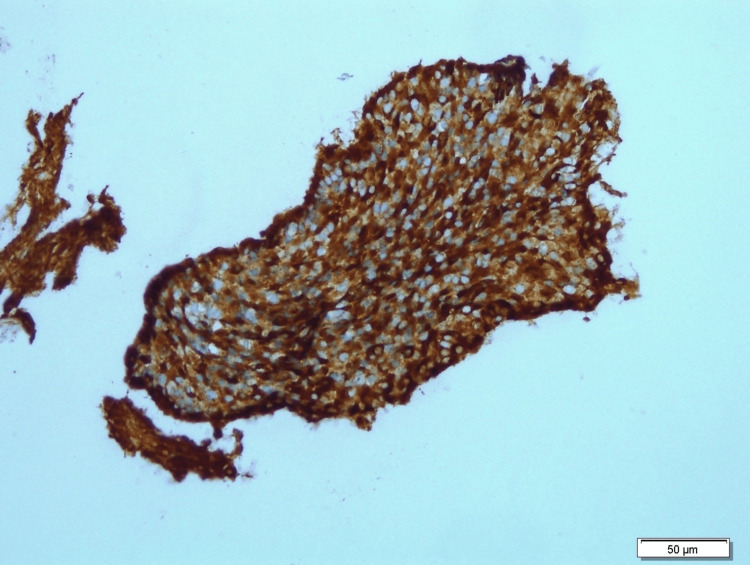
Immunohistochemistry study show the neoplastic cells are diffusely positive for CD56.

Examination of histological sections stained with hematoxylin and eosin revealed that tumor cells were arranged in sheets with a glandular and focal cribriform pattern (Figure 2). The tumor cells were round to polygonal in shape, with eosinophilic cytoplasm and oval to round nuclei displaying inconspicuous nucleoli. Nuclear pleomorphism was mild, and mitoses were observed at a rate of 0-1/2mm square. No necrosis, perineural invasion or lymphovascular invasion were detected. The neoplastic cells demonstrated diffuse positivity for CD56 (Figure 3). Although synaptophysin, chromogranin and Pan-CK immunostaining were performed interpretation of the result was not possible due to exhaustion of the neoplastic tissue materials. Sustentacular cells (S-100) were not identified, and the tumor cells exhibited a low proliferation index of less than 2% (Ki-67). Microscopic examination and immunohistochemistry confirmed the diagnosis of middle ear adenoma. The patient was also evaluated by the endocrine team for non-functioning middle ear adenoma.

**Figure 4:**
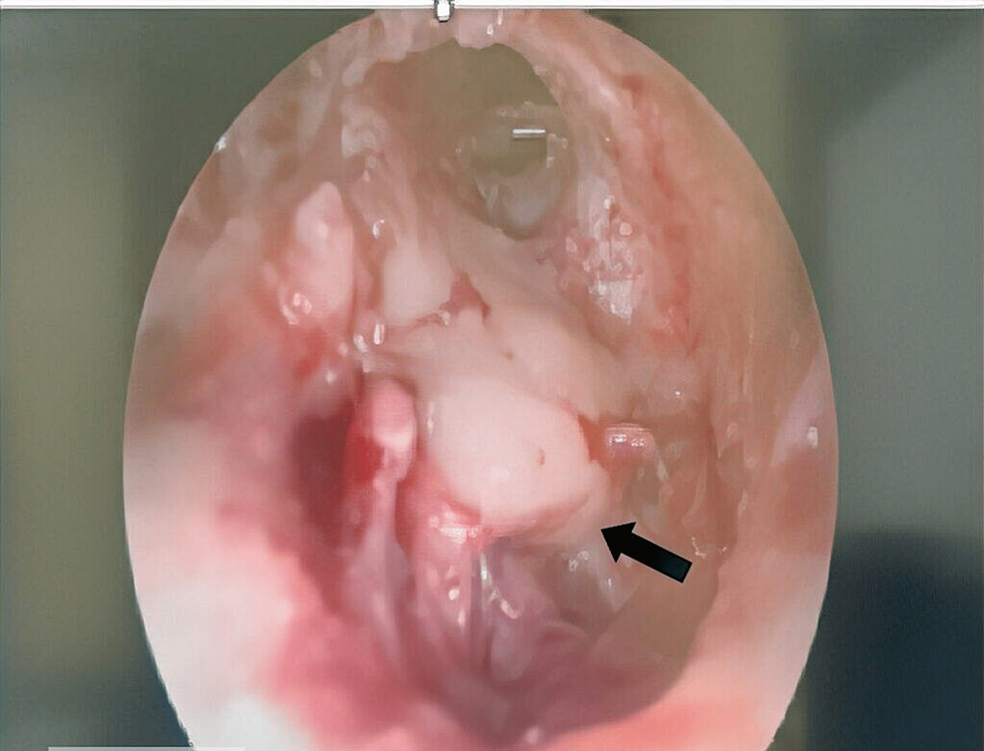
Intraoperative finding showing abnormal bony component at the anterior inferior part of left middle ear.

Postoperatively, the patient continued to experience persistent reduced hearing in the left ear, but there were no symptoms of otalgia, otorrhea or facial weakness. Otoscopy examination revealed tympanosclerosis, characterized by a dull tympanic membrane and a whitish opaque mass behind it. A staging CT scan conducted three months after the mastoidectomy exhibited a hypodensity in the middle ear cavity, with no significant bony erosion. This finding raised the possibility of residual or recurrent tumor. Consequently, a radical mastoidectomy was performed. During the surgery, an abnormal bony component was identified at the anterior inferior part of the middle ear (Figure 4). The lesion occupied the mesotympanum and was excised along with the tympanic membrane. All ossicles were intact, and there was no facial canal dehiscence. Histopathological examination confirmed the presence of adenoma with neuroendocrine differentiation. The patient is currently well and undergoing routine follow-up due to the risk of recurrence.

## Discussion

Head and neck NEN are a rare and diverse group of tumors. NEN in the middle ear are particularly uncommon, as epithelial cells with neuroendocrine features typically do not reside there [4]. The gastrointestinal tract and lungs are the most frequent locations for NEN [3].

There is no significant gender difference, and middle-ear neuroendocrine tumors (NETs) can occur across a wide age range without specific symptoms or findings. Typically, patients present with unilateral conductive hearing loss, ear fullness, tinnitus, and dizziness [9]. Consistent with this information, our patient exhibited a NET mimicking chronic otitis media, characterized by the presence of a mass behind the tympanic membrane, without facial paralysis or systemic symptoms. A thorough otoscopic examination, imaging studies, and biopsy are crucial for establishing an accurate diagnosis.

The differential diagnosis of middle ear NETs includes acoustic neuroma, rhabdomyosarcoma, jugulotympanic paraganglioma and adenocarcinoma [10]. Radiological diagnosis, including HRCT of the ear and MRI with a non-EPI-DWI protocol in case of a suspected middle ear tumor, is sufficient to establish a differential diagnosis of MEA. Imaging modalities such as HRCT and MRI should be performed preoperatively in order to exclude the diagnosis of paragangliomas, schwannomas, ceruminous gland adenomas, Schneiderian-type mucosal papillomas, lipomas, and cholesteatomas. Specifically, CT findings of neuroendocrine adenoma rarely show osteolytic destruction of the ossicles, which can be a helpful radiological sign in differentiating it from cholesteatoma and adenocarcinoma. Hybrid 111In Octreoscan SPECT/CT and PET/CT have higher sensitivity and resolution, and are crucial to confirm the results of the histopathological examination, in order to obtain an accurate differential diagnosis for this rare neoplasia [3]. In our case, SPECT/CT and PET/CT were not performed due to the unavailability of these modalities in our center. Paragangliomas are the most common tumoral lesions of the middle ear, and they can be differentiated histologically if they are negative for cytokeratin [11] . On MRI, middle ear NETs, appear isointense on T1-weighed images and close to hypointense on T2-weighted sequences (1). Acoustic neuroma typically presents as well circumscribed tumor with histology resembling schwannoma, characterized by fascicles, alternating hypocellular (Antoni B) and hypercellular (Antoni A) patterns, verocay bodies and diffuse positivity for S100, unlike MENET, which is negative for S100 [7].

Macroscopically, MENETs are typically unencapsulated and exhibit a soft, rubbery to firm consistency. They can appear as white, grey or red-brown in color and are usually smaller than 1 cm in size [7,12]. Microscopically, these tumors consist of exocrine and neuroendocrine cell types, sometimes expressing neuropeptides such as synaptophysin, serotonin, pancreatic polypeptides and chromogranin. The predominant cell morphology is cuboidal to columnar, with pale eosinophilic cytoplasm and round to oval nuclei displaying minimal pleomorphism. The chromatin pattern often exhibits a “salt and pepper” appearance, consistent with a neuroendocrine origin [13]. Saliba and Evrard classified middle ear glandular tumors into three subtypes: Type I, known as neuroendocrine adenoma of the middle ear (NAME), representing the most common subtype (76%) with positive immunohistochemistry and the absence of metastasis; Type II, middle ear adenoma (MEA) (20%) showing negative immunostaining and the absence of metastasis; and Type III, carcinoid tumor of the middle ear (CTME) (4%) characterized by positive immunostaining and the presence of metastasis and/or carcinoid syndrome [11]. The 2022 WHO Classification of Endocrine and NEN follows the IARC/WHO nomenclature framework and restricts the diagnostic term of “neuroendocrine carcinoma” to poorly differentiated epithelial NEN. Well-differentiated epithelial NEN are termed as NETs and are graded as follows: G1 NET (no necrosis and < 2 mitoses per 2 mm²; Ki67 < 20%), G2 NET (necrosis or 2-10 mitoses per 2 mm², and Ki67 < 20%) and G3 NET (> 10 mitoses per 2 mm² or Ki67 > 20%, and absence of poorly differentiated cytomorphology). Neuroendocrine carcinomas (> 10 mitoses per 2 mm², Ki67 > 20%, often associated with a Ki67 > 55%) are further subtyped based on cytomorphological characteristics as small cell and large cell neuroendocrine carcinomas [14].

Functional NETs typically manifest with symptoms related to the overproduction of hormones by the neoplasm, such as insulinoma, glucagonoma and VIPoma. Diagnosis of these tumors can be achieved through mono analyte testing. However, for non-functional tumors, there are currently no universally available biomarkers [15]. In our case, postoperative blood investigations during routine follow up would not yield significant findings, as the patient is asymptomatic and has been diagnosed with a non-functioning NET.

Surgery, with or without removal of ossicles, is the treatment approach for this rare condition. Previous reports have indicated a local recurrence rate of 12.7%, which may necessitate additional surgical intervention. Radiation, chemotherapy and somatostatin analogues are typically employed in the management of pulmonary or gastrointestinal carcinoids [16].

## Conclusions

Middle-ear adenomas are rare neoplasm. Surgical resection is typically the primary treatment, and microscopic, and immunohistochemical examinations are necessary for a definitive diagnosis of middle ear NETs. Long-term follow-up is recommended to monitor for residual tumors, late recurrence, and metastatic disease. It is crucial for ENT surgeons to not only consider chronic otitis media in patients with frequent discharge but also to investigate and rule out the possibility of tumors.
